# Interspecific competition among catch crops modifies vertical root biomass distribution and nitrate scavenging in soils

**DOI:** 10.1038/s41598-019-48060-0

**Published:** 2019-08-08

**Authors:** Diana Heuermann, Norman Gentsch, Jens Boy, Dörte Schweneker, Ulf Feuerstein, Jonas Groß, Bernhard Bauer, Georg Guggenberger, Nicolaus von Wirén

**Affiliations:** 1 Molecular Plant Nutrition, Leibniz Institute of Plant Genetics and Crop Plant Research Gatersleben, Corrensstraße 3, 06466 Stadt Seeland, Germany; 2 Institute of Soil Science, Leibniz Universität Hannover, Herrenhäuser Straße 2, 30419 Hannover, Germany; 3 Deutsche Saatveredelung AG, Steimker Weg 7, 27330 Asendorf, Germany; 4 Crop Production and Crop Protection, Hochschule Weihenstephan-Triesdorf, Steingruberstraße 2, 91746 Weidenbach, Germany; 5 Present Address: Kuratorium für Technik und Bauwesen in der Landwirtschaft e.V., Bartningstraße 49, 64289 Darmstadt, Germany

**Keywords:** Plant ecology, Agroecology

## Abstract

The potential of a plant species to acquire nutrients depends on its ability to explore the soil by its root system. Co-cultivation of different species is anticipated to lead to vertical root niche differentiation and thus to higher soil nutrient depletion. Using a qPCR-based method we quantified root biomass distribution of four catch crop species in vertical soil profiles in pure vs. mixed stands. Pure stands of mustard and phacelia robustly reached 70 cm soil depth, while oat preferably colonized upper soil layers, and clover developed the shallowest and smallest root system. Analysis of residual nitrate pools in different soil depths and correlation with root biomass showed that, besides rooting depth also root biomass determines soil nitrogen depletion. While occupying the same vertical niches as in pure stands, mustard and phacelia dominated total root biomass of the mix. In contrast, root biomass of clover and oat was severely suppressed in presence of the other species. Below-ground biomass profiling indicated low niche complementarity among the root systems of the examined species. Nonetheless, the mixture mostly overyielded root biomass of the pure stands and thus shows higher potential for efficient soil exploration by roots.

## Introduction

Efficient nutrient acquisition of a plant species depends to a large extent on its root system architecture^1^. In particular deep rooting is crucial for exploiting subsoil water or nutrient reserves and scavenging of mobile nutrient forms. Investigating root frequency distributions at different soil depths in a wide range of species, Thorup-Kristensen^2,3^ found that deeper rooting species were more efficient in nitrogen (N) acquisition than shallow-rooting ones, probably as N in the form of nitrate is highly prone to leaching into deeper soil layers^3^. Most effective species were crucifers, namely fodder radish or winter rape, whose roots can reach down to more than 2 m soil depth^2–5^. Especially when topsoils fall dry or are depleted of nutrients, subsoils can provide up to two third of the N required by plants^6^. With regard to phosphorus (P) acquisition, however, species or genotypes with shallow root angles have been identified as highly efficient, because the majority of the readily available P is usually located in the topsoil layer^7,8^. There, physiological mechanisms come into play that improve P acquisition and include the release of protons, organic acids, P-mobilizing enzymes and the ability to establish symbioses with mycorrhizae^9,10^. Considering the fact that individual plant species tend to form root systems with either steep or shallow root angles that are beneficial for nutrient acquisition from either deep or top soil layers, respectively, the combination of different species in a mixture is regarded as valuable tool for efficient nutrient recovery and sustainable nutrient management in agricultural crop rotations^11^. Catch crops are grown over winter or other periods with poor vegetation with the aim of conserving nutrients for the subsequent release to the following crop in order to reduce fertilizer inputs^12^. Catch crops can scavenge substantial amounts of soil N^13^ or mobilize P and release it in plant-available forms to the following crop during the mineralization of their biomass^10,14^. Thereby, leaching of soluble nutrient forms during rainfalls or thawing events can be decreased^15^. This is of high importance as, once below the rooting zone, nitrate transfer into the hydrosphere can cause severe environmental problems, such as eutrophication or hypoxia^16^. To what extent the cultivation of a species mixture with different root architectures can reduce N and P pools in the soil relative to pure stands of catch crops is so far poorly studied.

Mixtures of catch crop species may enhance nutrient retention by exploiting a larger soil volume. Functional complementarity within mixtures may rely on vertical root niche differentiation, whenever the rooted soil volume is more efficiently explored^17,18^. However, determining root distribution and assigning root biomass to individual species remains challenging^18,19^. Mainly due to the lack of proper root quantification techniques, studies relating nutrient uptake by catch crops to their root growth were conducted with single species only (e.g.^2,5,20,21^). Since higher-diversity mixtures can outperform productivity of pure stands^18^, studying niche differentiation of roots in mixtures together with soil nutrient scavenging within vertical soil profiles may allow evaluating the importance of root biomass for nutrient depletion in soils.

So far, most studies describing root architectural traits have been conducted with single species grown on agar, in hydroponics or in rhizotrons, where root systems are easily accessible (e.g.^22–25^). Alternatively, x-ray computer tomography (µ-CT) has been refined to monitor root architectural changes over time in a non-destructive way^26^. To enable root phenotyping of field-grown plants, imaging of excavated topsoil root systems have been combined with algorithmic approaches allowing to simulate root traits in deeper soil layers^27^. However, also with these advanced methods capturing root traits of mature plants in deeper soil horizons remains challenging, especially when plant species grow in mixtures. In order to distinguish and quantify root biomass from individual plant species grown in soils with multi-species mixtures, DNA-based methods appear to be a straightforward way^28,29^. By targeting poorly conserved DNA regions, Mommer, *et al*.^30^ developed a qPCR-based protocol using species-specific DNA fragments for species-specific DNA amplification. With this method the authors showed that in a four-species mixture consisting of two dicots and two grasses one dicot species strongly increased topsoil root mass density in the mixture relative to pure stands^17^. As interspecific interactions strongly depend on species combinations^31^, the question arises whether catch crop mixtures explore the vertical soil profile in a complementary way and whether they can deplete more nutrients by exploiting nutrient pools at different depths.

The present study aimed at determining root biomass distribution along vertical soil profiles in four catch crop species that were anticipated to differ in root growth properties. We hypothesized that co-cultivation of different species in a mixture promotes niche complementarity of root systems and allows more efficient soil N and P depletion compared to pure stands. We further anticipated that root biomass formation is as important as rooting depth for efficient nutrient depletion from deeper soil layers. Therefore, deep-rooting white mustard (*Sinapis alba*) or Egyptian clover (*Trifolium alexandrinum*) as well as shallower rooting lacy phacelia (*Phacelia tanacetifolia)* or bristle oat (*Avena strigosa*)^32^ were cultivated in pure culture or in a four-species mix at two field sites in Germany. DNA-based root biomass quantification via qPCR in soil samples from different soil depths allowed reconstructing and comparing root biomass profiles in vertical orientation. In parallel, we analyzed N and P pools in different soil depths to determine the influence of root biomass distribution on the depletion of these nutrient pools.

## Results

### Root niche differentiation in pure and mixed cultures

**Figure 1:**
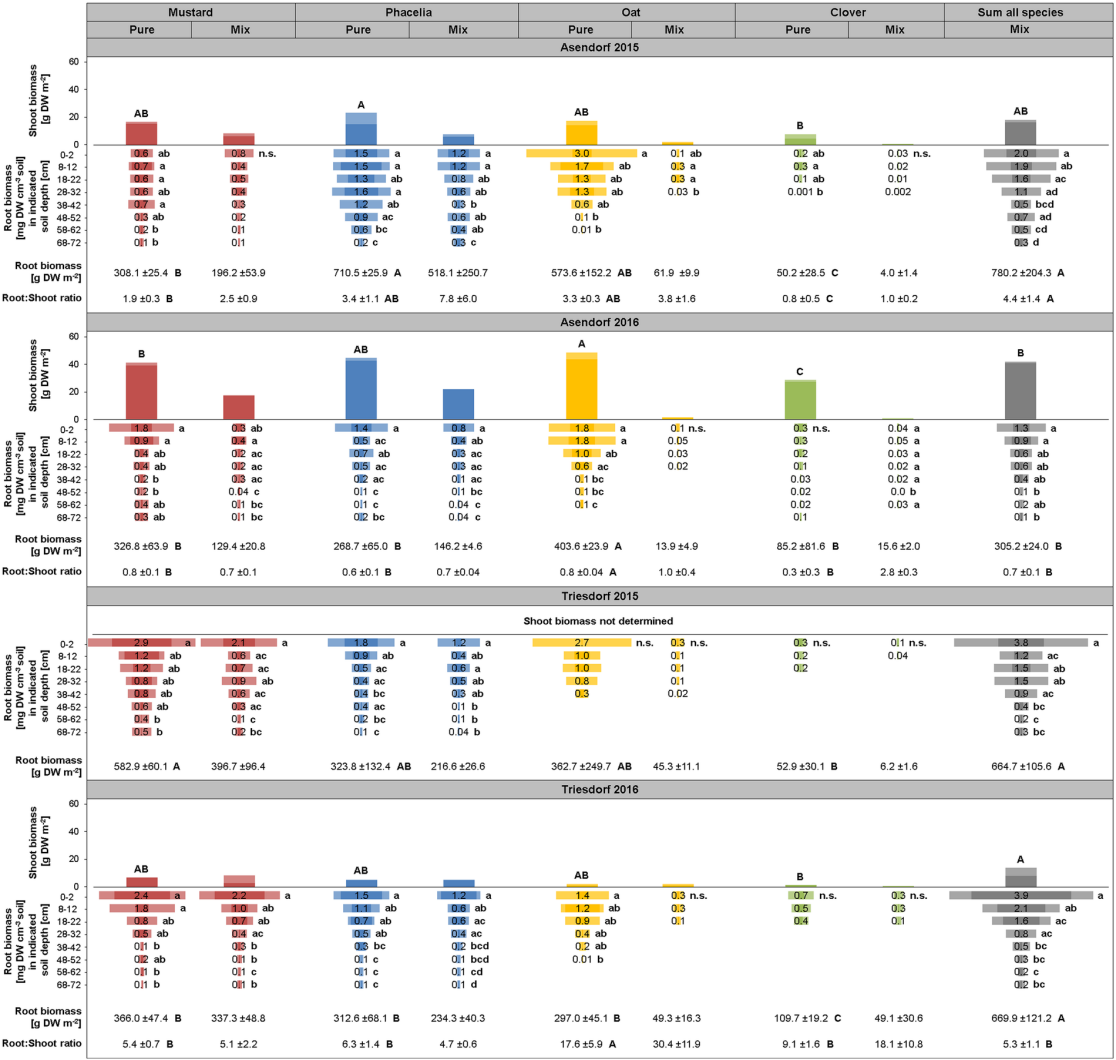
Root biomass in different soil depths, total root and shoot biomass as well as root:shoot ratio in 4 catch crop species in pure stands vs. mixed cultivation in 2 locations and years. Vertical bars show means of shoot biomass -s.d. (pale colour); n = 3. Horizontal bars show means (indicated by central numbers) of root biomass according to qPCR-based quantification −0.5 s.d. from every side of the bar; n = 7–9. “Root biomass” below horizontal bars shows means of total root biomass down to ~70 cm soil depth ± s.d.; n = 7–9. Therefore, root biomasses [mg DW cm^−3^ soil] were interpolated, summed and scaled up to 0.7 m^−3^ soil volume. “Root:Shoot ratio” shows means of root biomass [g DW m^−2^] to shoot biomass [g DW m^−2^], n = 3. Upper case letters: Differences among total root biomass, shoot biomass or root:shoot ratio among the 5 cultivated catch crop variants (Pure stands of mustard, phacelia, oat and clover and the 4-species mixture) according to Tukey’s test at p < 0.05. Lower case letters: Differences among root biomasses in different soil depths of one species in pure or mixed cultivation according to Tukey’s test on ranks at p < 0.05.

We first addressed the question whether root systems of purely grown catch crops occupy different niches in the vertical soil profile at the end of the catch crop cultivation period. At both sites and in both years, mustard and phacelia developed the deepest root systems, reaching down to at least 70 cm (Fig. 1). Mustard generally developed a larger root biomass in the topsoil at the site Triesdorf, while in Asendorf it poorly explored the topsoil, especially in 2015. This was probably not due to unfavourable growth conditions in the topsoil, because there phacelia and oat rooted extensively in the topsoil. In the other three environments, both species produced less root biomass, which coincided with lower precipitation and soil water contents (Table 1, Supplementary Table S-1). Despite varying root depth, oat showed most consistently a coherent vertical root biomass profile down to 20–30 cm depth (Fig. 1), suggesting this species to proliferate roots preferably in the topsoil. Root biomass formation of clover was smallest, and roots hardly exceeded 30 cm soil depth. Taken together, vertical root biomass differentiation was mainly shaped by environmental conditions, whereas maximum root depth appeared to be determined more by the species themselves.

**Table 1:** Weather conditions during the experimental runtime at the sites Asendorf and Triesdorf as recorded by local weather stations.

	Asendorf		Triesdorf	
	2015	2016	2015	2016
*Avg. Temperature [°C]*				
Aug	19.2	17.6	20.2	17.8
Sep	13.4	17.6	12.6	16.1
Oct	8.9	9.1	8.0	7.5
Nov	8.4	4.2	6.4	3.0
*Precipitation [mm]*				
Aug	135.0	20.6	34.5	38.0
Sep	55.4	29.6	20.3	39.1
Oct	63.2	29.4	52.3	54.8
Nov	175.4	66.4	77.0	52.5

We then investigated whether species- and environment-specific differences in vertical root biomass distribution were also maintained in the four-species mix. To a large extent mustard and phacelia maintained the same vertical root profiles as in pure stands (Fig. 1). Interestingly, especially in the topmost soil layer root biomass of both species was mostly as large as in their pure stands, although much lower biomass was expected due to lower stand densities in the mix relative to pure stands (Table 2). Against this, root biomass of oat and clover was reduced to <20% in mixed cultivation. While clover showed highly similar vertical root biomass distribution as in pure stands, oat failed to explore the topsoil and fairly developed beyond 30 cm soil depth (Fig. 1). Clearly, mustard and phacelia dominated root biomass in the mix with a share of 50–60% by mustard in Triesdorf and 50–65% by phacelia in Asendorf. Thus, not only the low biomass-producing species clover but also oat suffered severely from a suppressive effect on root development by the vigorously growing species.

**Table 2:** Conditions of catch crop cultivation at the experimental stations in Asendorf and Triesdorf.

	Asendorf	Triesdorf		
	2015	2016	2015	2016
*Stand properties*				
CC seeding date	03/09/2015	22/08/2016	20/08/2015	24/08/2016
Plot size	7.3 m × 6 m		7.3 m × 6 m	
*Seeding rate pure stands [seeds m*^*−*2^]				
Mustard	300		300	
Phacelia	706		706	
Oat	588		588	
Clover	833		833	
*Seeding rate mix [seeds m*^*−*2^]				
Mustard	67		67	
Phacelia	294		294	
Oat	53		53	
Clover	233		233	
Mix total	647		647	
*Stand density pure stands [plants m*^*−*2^]				
Mustard	303.9 ± 107.2	n.a.	240.9 ± 19.9	198.7 ± 102.5
Phacelia	509.0 ± 95.6	n.a.	333.3 ± 22.2	271.2 ± 55.3
Oat	438.5 ± 23.4	n.a.	348.4 ± 9.2	150.5 ± 20.1
Clover	579.5 ± 96.8	n.a.	268.9 ± 23.9	294.4 ± 43.9
*Stand density mix [plants m*^*−*2^]				
Mustard	62.8 ± 19.9	n.a.	61.3 ± 5.2	57.0 ± 7.0
Phacelia	61.5 ± 5.6	n.a.	134.7 ± 16.0	102.6 ± 16.0
Oat	69.8 ± 5.4	n.a.	33.3 ± 7.8	14.5 ± 3.2
Clover	52.8 ± 17.6	n.a.	84.0 ± 5.9	41.5 ± 24.5
Mix total	244.9 ± 56.0	n.a.	313.3 ± 15.1	215.7 ± 15.5

CC = catch crop. n.a. = not analysed. Stand density shows mean ± s.d.; n = 3.

The below-ground species-specific competition in the mix was reflected also in above-ground biomass. In Triesdorf, mustard contributed 60% to total shoot biomass of the mix, while in Asendorf phacelia held a share of 50–60% (Fig. 1). Oat and clover contributed barely to shoot biomass of the mix. However, in these two species the root:shoot ratio tended to increase when cultivated in mixture with the other species, indicating that competition favoured their root over shoot growth. Root:shoot ratios of mustard and phacelia were lower and not consistently influenced by mixed cultivation, but a tendency for decreased root:shoot ratios from pure stands to mix in Triesdorf 2016, when catch crop stands were loose due to drought (Table 2), suggested that mustard and phacelia outcompeted the other species mainly by more efficient soil and resource exploration.

### Assessment of soil nitrogen and phosphate pools in relation to root biomass distribution

**Figure 2:**
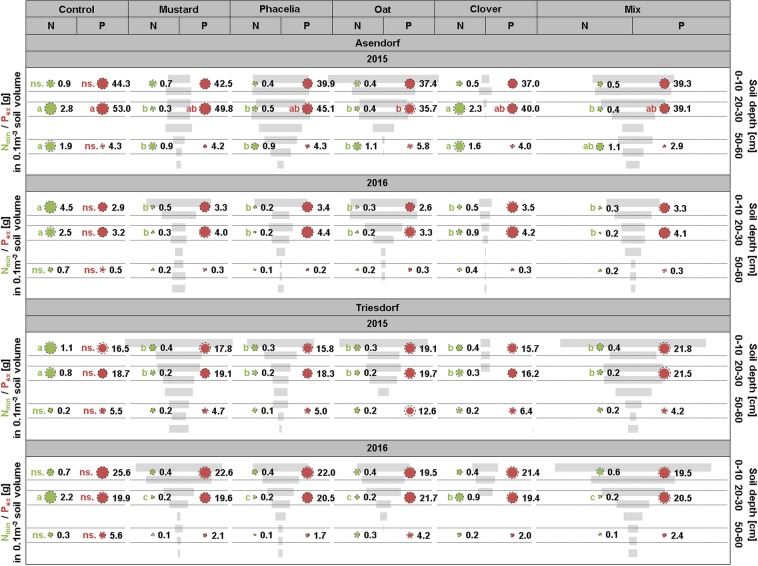
Soil pools of N_min_ and P_ex_ in different soil depths in relation to root biomass distribution of catch crop variants in 2 locations and years. Bubble areas represent N (green) or P (red) pool sizes and show means + s.d. (dashed lines); n = 3. Control represents fallow plots. Grey bars represent total root biomass of individual or mixed catch crop variants in different soil depths (referring to Fig. 1). Letters: Differences among soil N_min_ or P_ex_ pools within one depth according to Tukey’s test on ranks at p < 0.05; ns. = not significant.

To determine the influence of root system distribution of the five catch crop variants on pool sizes of available N and P in the soil, N_min_ as well as P_ex_ were determined in two topsoil segments and in the 50–60 cm segment, representing a snapshot of nutrient pools at the time of root sampling. As evident from soil analysis in the fallow (control), N appeared only to be relocated to deeper soil layers in Asendorf 2015 when rainfalls were highest (Fig. 2, Table 1), whereas other relevant soil parameters (cation exchange capacity, organic carbon, water holding capacity) did not differ between the locations. Significant N_min_ depletion depended on a species’ rooting depth. When in Asendorf 2015 clover roots failed to efficiently develop biomass in 20–30 and in 50–60 cm depth, corresponding N_min_ pools were not depleted. Under the same conditions, oat formed rather little root biomass below 40 cm but nevertheless depleted N_min_ pools significantly. This raised the question to what extent N depletion correlates with root biomass in the respective soil depths.

We first itemized N_min_ into NO_3_^−^ and NH_4_^+^ and found up to 45× larger NO_3_^−^ than NH_4_^+^ pools in soil segments of control treatments (Table 3). However, in root-containing segments this ratio decreased due to NO_3_^−^ depletion, which showed a similar pattern as observed for N_min_, whereas NH_4_^+^ pool sizes were hardly influenced by any of the catch crop variants. An exception were increased NH_4_^+^ pools under mustard in the topsoil in Asendorf, suggesting that this species enhanced ammonification or retarded nitrification.

**Table 3:** Pool sizes of NO_3_^−^ and NH_4_^+^ in different soil depths of the individual catch crop variants.

	Control	Mustard	Phacelia	Oat	Clover	Mix
***Asendorf 2015***						
*NO*_3_^*−*^*-N [g]*						
0–10 cm	0.68 ± 0.10^**a**^	0.26 ± 0.13^**b**^	0.19 ± 0.09^**b**^	0.23 ± 0.12^**b**^	0.33 ± 0.07^**b**^	0.22 ± 0.10^**b**^
20–30 cm	2.66 ± 0.14^**a**^	0.17 ± 0.09^**b**^	0.21 ± 0.07^**b**^	0.19 ± 0.09^**b**^	2.14 ± 0.21^**a**^	0.20 ± 0.04^**b**^
50–60 cm	1.87 ± 0.73^**a**^	0.75 ± 0.24^**c**^	0.89 ± 0.10^**c**^	1.03 ± 0.23^**b**^	1.55 ± 0.66^**ab**^	0.97 ± 0.26^**c**^
*NH*_4_^+^*-N [g]*						
0–10 cm	0.22 ± 0.21^**ns**.^	0.45 ± 0.31	0.18 ± 0.07	0.21 ± 0.22	0.14 ± 0.14	0.25 ± 0.02
20–30 cm	0.13 ± 0.17^**ns**.^	0.17 ± 0.13	0.24 ± 0.24	0.21 ± 0.15	0.19 ± 0.27	0.21 ± 0.10
50–60 cm	0.07 ± 0.09^**ns**.^	0.15 ± 0.13	0.04 ± 0.07	0.07 ± 0.06	0.08 ± 0.14	0.11 ± 0.06
***Asendorf 2016***						
*NO*_3_^*−*^*-N [g]*						
0–10 cm	4.45 ± 0.44^**a**^	0.16 ± 0.04^**b**^	0.04 ± 0.02^**b**^	0.08 ± 0.07^**b**^	0.24 ± 0.13^**b**^	0.06 ± 0.03^**b**^
20–30 cm	2.36 ± 1.08^**a**^	0.13 ± 0.09^**b**^	0.09 ± 0.07^**b**^	0.06 ± 0.06^**b**^	0.66 ± 0.26^**b**^	0.01 ± 0.01^**b**^
50–60 cm	0.58 ± 0.18^**ns**.^	0.14 ± 0.06	0.04 ± 0.05	0.15 ± 0.10	0.37 ± 0.25	0.07 ± 0.06
*NH*_4_^+^*-N [g]*						
0–10 cm	0.08 ± 0.07^**b**^	0.31 ± 0.01^**a**^	0.19 ± 0.04^**ab**^	0.20 ± 0.03^**ab**^	0.26 ± 0.10^**b**^	0.22 ± 0.04^**ab**^
20–30 cm	0.11 ± 0.05^**ns**.^	0.12 ± 0.03	0.11 ± 0.04	0.16 ± 0.02	0.20 ± 0.02	0.15 ± 0.01
50–60 cm	0.11 ± 0.09^**ns**.^	0.09 ± 0.08	0.11 ± 0.01	0.04 ± 0.07	0.07 ± 0.06	0.11 ± 0.01
***Triesdorf 2015***						
*NO*_3_^*−*^*-N [g]*						
0–10 cm	1.05 ± 0.23^**a**^	0.27 ± 0.01^**b**^	0.26 ± 0.03^**b**^	0.24 ± 0.08^**b**^	0.29 ± 0.05^**b**^	0.31 ± 0.05^**b**^
20–30 cm	0.70 ± 0.24^**a**^	0.11 ± 0.03^**b**^	0.11 ± 0.05^**b**^	0.015 ± 0.10^**b**^	0.30 ± 0.12^**b**^	0.09 ± 0.03^**b**^
50–60 cm	0.21 ± 0.01^**a**^	0.05 ± 0.01^**c**^	0.08 ± 0.06^**b**^	0.19 ± 0.01^**a**^	0.18 ± 0.05^**ab**^	0.06 ± 0.04^**c**^
*NH*_4_^+^*-N [g]*						
0–10 cm	0.10 ± 0.10^**ns**.^	0.11 ± 0.02	0.07 ± 0.06	0.05 ± 0.05	0.07 ± 0.04	0.08 ± 0.03
20–30 cm	0.06 ± 0.06^**ns**.^	0.07 ± 0.07	0.08 ± 0.02	0.02 ± 0.04	0.02 ± 0.04	0.13 ± 0.07
50–60 cm	0.03 ± 0.01^**ns**.^	0.10 ± 0.07	0.03 ± 0.02	0.06 ± 0.06	0.03 ± 0.05	0.11 ± 0.02
***Triesdorf 2016***						
*NO*_3_^*−*^*-N [g]*						
0–10 cm	0.54 ± 0.11^**a**^	0.26 ± 0.02^**b**^	0.25 ± 0.07^**b**^	0.66 ± 0.37^**a**^	0.32 ± 0.06^**b**^	0.39 ± 0.22^**a**^
20–30 cm	2.15 ± 0.20^**a**^	0.13 ± 0.02^**c**^	0.13 ± 0.03^**c**^	0.17 ± 0.05^**c**^	0.90 ± 0.21^**b**^	0.14 ± 0.06^**c**^
50–60 cm	0.30 ± 0.04^**ns**.^	0.05 ± 0.05	0.05 ± 0.03	0.27 ± 0.11	0.21 ± 0.04	0.07 ± 0.03
*NH*_4_^+^*-N [g]*						
0–10 cm	0.16 ± 0.07^**ns**.^	0.15 ± 0.05	0.11 ± 0.05	0.10 ± 0.06	0.11 ± 0.03	0.16 ± 0.09
20–30 cm	0.05 ± 0.02^**ns**.^	0.04 ± 0.03	0.06 ± 0.04	0.05 ± 0.06	0.04 ± 0.05	0.01 ± 0.01
50–60 cm	0.03 ± 0.02^**ns**.^	0.02 ± 0.02	0.02 ± 0.02	0.07 ± 0.06	0.03 ± 0.03	0.02 ± 0.02

Samples of 0.1 m^−3^ were taken from 3 plots at 3 different soil depths for extraction of nutrient pools. Numbers show means ± s.d., n = 3. Control represents fallow plots. Different letters indicate significant differences among soil NO_3_^−^ or NH_4_^+^ pools of individual catch crop variants at one depth according to Tukey’s test on ranks at p < 0.05; ns. = not significant.

We then calculated correlations between root biomass and nutrient depletion within soil layers and observed mostly no quantitative relation between actual root biomass in a soil segment and recorded depletion of NO_3_^−^ pools (Table 3). However, in Triesdorf NO_3_^−^ depletion in the deepest segment, i.e. 50–60 cm, correlated significantly with catch crop root biomass, even though NO_3_^−^ pool sizes were rather low (Table 3). Remarkably, in Asendorf 2016 NO_3_^−^ depletion was significantly associated with root biomass in 20–30 cm depth.

At both locations, pool sizes of P_ex_ were similar in the upper two soil layers but 3–12 times larger than in the deepest segment (Fig. 2), reflecting the low mobility of P in soils^10^. In general, P pools were not significantly affected by the root biomass distribution of catch crops. Only in Asendorf 2015, where topsoil P_ex_ pools were largest among all environments, oat significantly depleted P_ex_ in 20–30 cm compared to fallow (Fig. 2). In this particular case, root biomass itself was unlikely the cause for P depletion as phacelia roots proliferated similar vigorously in the same soil layer.

## Discussion

Root system architecture is considered a major morphological trait determining the nutrient foraging capacity of a plant species^1^. This view is mainly based on the observation that mild deficiencies of certain nutrients, especially of N and P, enhance lateral root proliferation^22^, and is supported by correlative evidence between root frequency distributions and nutrient depletion in different soil depths or nutrient accumulation in shoots^3,21^. Beyond this plant nutritional perspective, extensive root exploration of soils fixes nutrients in the shoot and root biomass, preventing them from leaching^15,33^. To compare vertical root biomass profiles of different catch crops and investigate their role in soil nutrient depletion, we took a novel approach by correlating root biomass distribution with nutrient pool sizes at different soil depths. This approach revealed that species-dependent root biomass profiles are only partly maintained when grown in mixed versus pure culture and that besides rooting depth the quantity of the root biomass can be of particular importance for nitrate depletion.

Nitrate is highly prone to leaching into the subsoil^34^. This was particularly relevant in Asendorf 2015 where predominantly higher rainfalls translocated nitrate to deeper soil layers (Table 1, Table 3). There, only mustard, phacelia and oat significantly depleted nitrate pools at 20–30 and 50–60 cm depth, while clover roots hardly reached the 20 cm layer and thus even failed exploiting the corresponding nitrate pool (Fig. 1, Table 3). The origin of Egyptian clover from warmer climates of Syria and Egypt^35^ may explain its slower development and thus the comparably lower biomass reached at the end of the cultivation period. Topsoil root biomass was most likely sufficient or in excess of the minimum biomass required for nitrate depletion (Table 4). This view is supported by the observation that even the small root biomass produced by clover in the topsoil in the other three environments was sufficient to effectively deplete the corresponding nitrate pool (Fig. 1, Table 3), even though clover is less dependent on soil N uptake due to N fixation^36^. Earlier studies installing glass tubes in soils to count root intersections consistently found that root depth rather than root intensity, i.e. root abundance per areal unit, correlated with residual subsoil nitrate^2–5^. In contrast to these reports, we found in the deepest investigated soil segment in Triesdorf an association between nitrate depletion and root biomass, while in Asendorf, especially in 2016, such correlation was present in the 20–30 cm topsoil layer (Table 4). Notably, exactly these three soil segments had the lowest water contents within the vertical soil profile (Supplementary Table S-1). We therefore concluded that exploitation of nitrate pools within a certain soil segment relies not only on the presence of roots but also on their quantity, especially when mass flow of nitrate to the root is impaired by low water availability.

**Table 4:** Correlations between the depletion of soil N or P fractions and root biomasses of catch crops in the same soil segments.

	Root biomass [g DW 0.1 m^−3^ soil volume]			
N/P pool depletion in 0.1 m^−3^ soil vol.	Asendorf 2015	Triesdorf 2015	Asendorf 2016	Triesdorf 2016
**0–*10 cm***				
N_min_ [g]	r = 0.14, p = 0.62	**r = 0.19, p = 0.50**	r = 0.35, p = 0.19	r = −0.06, p = 0.81
NO_3_^−^ [g]	r = 0.35, p = 0.21	**r = 0.45, p = 0.09**	r = 0.49, p = 0.06	r = −0.04, p = 0.89
NH_4_^+^ [g]	r = −0.02, p = 0.94	**r = −0.28, p = 0.31**	**r = 0.22, p = 0.43**	**r = −0.41, p = 0.13**
P_ex_ [kg]	r = −0.15, p = 0.58	r = 0.03, p = 0.91	**r = 0.34, p = 0.22**	**r = 0.33, p = 0.24**
**20–*30 cm***				
N_min_ [g]	r = 0.33, p = 0.22	r = 0.06, p = 0.81	r = 0.56, p = 0.02	r = 0.63, p = 0.01
NO_3_^−^ [g]	r = 0.44, p = 0.10	r = 0.39, p = 0.14	r = 0.65, p < 0.01	r = 0.52, p = 0.04
NH_4_^+^ [g]	**r = −0.17, p = 0.56**	**r = −0.40, p = 0.14**	**r = 0.27, p = 0.32**	r = 0.27, p = 0.33
P_ex_ [kg]	**r = 0.30, p = 0.28**	**r = −0.42, p = 0.12**	**r = 0.18, p = 0.51**	**r = −0.33, p = 0.22**
**50–*60 cm***				
N_min_ [g]	r = 0.26, p = 0.34	r = 0.48, p = 0.06	r = 0.06, p = 0.81	r = 0.81, p < 0.001
NO_3_^−^ [g]	r = 0.23, p = 0.29	r = 0.87, p < 0.001	r = 0.07, p = 0.78	r = 0.79, p < 0.001
NH_4_^+^ [g]	r = −0.11, p = 0.70	r = −0.43, p = 0.10	r = −0.37, p = 0.17	r = 0.43, p = 0.10
P_ex_ [kg]	r = 0.15, p = 0.58	r = 0.15, p = 0.59	r = −0.09, p = 0.75	r = −0.09, p = 0.73

N or P pool depletion was calculated by subtracting pool sizes under catch crops from the mean of the respective pool size in the fallow (referring to Fig. 2 and Table 3). Root biomasses were interpolated from values shown in Fig. 1 and scaled up to 0.1 m^−3^ soil volume of the indicated segments. Pearson product moment (black) or Spearman rank order correlations (grey) include all 5 catch crop variants; n = 15.

The pool sizes of ammonium were several fold lower than those of nitrate and decreased from the topsoil in fallow treatments mostly to approx. 30% in 50–60 cm depth (Table 3). However, none of the catch crops succeeded to significantly deplete these ammonium pools, probably because ammonium uptake was compensated for by ammonium replenishment from mineralized organic matter or desorption. Instead, ammonium pools in the upper soil layer in Asendorf even increased under mustard. This may point to root exudation of biological nitrification inhibitors. Indeed, members of the Brassicaceae are known to release glucosinolates with their root exudates^37^, and their degradation products can act against nitrifying bacteria in the soil^38^.

Due to its low mobility, phosphate is also mostly located in the topsoil and has to reach the root surface mainly via diffusion^10^. Consequently, shallow root systems and abundant topsoil root proliferation are beneficial for phosphate acquisition^8^. Against the expectation that catch crops with abundant topsoil rooting deplete phosphate pools more efficiently, there was no consistent influence of root biomass distribution on the depletion of investigated soil P_ex_ pools (Fig. 2). Eichler-Löbermann, *et al*.^14^ found that among five catch crop species phacelia most efficiently increased soil P contents and subsequent P uptake by the following crop, indicating a strong positive effect on soil P mobilization apart from an elevated P uptake capacity of phacelia. In the present study, P_ex_ pools under phacelia were not depleted (Fig. 2), suggesting that root-absorbed P was replenished by de-novo mobilization, as supposed in another case^39^. Like phacelia, also oat has been previously described to form a shallow root system^32^ and was indeed among the catch crop species with the largest topsoil root biomass in our study (Fig. 1). Oat was the only species in our experiment depleting significantly P_ex_ in 20–30 cm depth in Asendorf 2015, where water supply from rainfalls was largest among all environments (Fig. 2, Table 1, Supplementary Table S-1). These moist conditions may have favoured conversion of the stable to the available P fraction in the soil^40^ and subsequent P uptake from the latter pool. This notion is supported by the observation that next to oat also all other catch crop species tended to decrease soil P_ex_ in that environment (Fig. 2). However, to what extent fine roots or root hairs contributed to total root biomass of each species could not be unlocked with our qPCR-based method of root biomass determination. It thus remains open whether superior P_ex_ depletion in oat profited from a higher root surface to root biomass ratio relative to the other species.

Spatial niche differentiation of root systems plays a critical role to stabilize species communities in ecosystems^41^. When combining four catch crop species with formerly described differences in root system distribution in a mixture, mustard and phacelia occupied the same vertical root niches as they did in pure stands. In contrast, oat and clover lost their vertical niche differentiation; clover suffered mainly from lower biomass but maintained rooting depth, while oat roots decreased both, biomass and rooting depth (Fig. 1). Thus, in each environment these two species appeared to be outcompeted from their root niches by mustard and phacelia, irrespective of established stand densities, which did not lead to proportional differences in root biomass (Table 2, Fig. 1). Indeed, their fast growth and high nutrient uptake characterize mustard and phacelia as competitive in nutrient-rich environments as in agricultural soils, in which light is one of the most limiting resources^42^. This suggests a lower assertiveness of oat and clover for soil exploration in certain multi-species combinations. At the date of harvest, mustard and phacelia were most advanced in development (see 4.2) suggesting that they are most adapted to autumn climates in Germany. This may have contributed also to their high competitiveness. Notably, only when mustard and phacelia performed well in the mix total root biomass of the mix outperformed that of each pure stand (Asendorf 2015, Triesdorf 2015 and 2016; Fig. 2). This suggested that only the combination of mustard and phacelia synergistically increased root biomass formation in the mix. Of course, these results depend to some extent on seeding ratios. Testing additional ratios of component species by serial replacement studies may further increase biomass yield of the mix^43^. Although superior root biomass of the mix did not translate into higher nutrient depletion - at least in the snapshot we obtained at the end of the cultivation period (Fig. 2) - it confers robustly a larger potential for root-bound nutrient retrieval and conservation in the topsoil compared to pure stands, which is favourable for reducing nutrient losses and improving nutrient carry-over to the following crop after mineralization.

Taken together, the present analysis of vertical root biomass profiles indicates rather low niche complementarity among the root systems of the four chosen species. Instead, vertical root biomass distribution in mixed cultivation was characterized by inter-species competition but also by synergism between species, promoting overall soil exploration by roots. The fact that such opposing below-ground effects were not necessarily reflected in above-ground biomass formation emphasizes the need to further explore inter-species compatibility also at the root level.

## Materials and Methods

### Plant material and growth conditions

Four catch crop species were grown: White mustard (*Sinapis alba*) cv. Litember, lacy phacelia (*Phacelia tanacetifolia*) cv. Bee Happy, bristle oat (*Avena strigosa*) cv. Panache and Egyptian clover (*Trifolium alexandrinum*) cv. Alex.

#### Greenhouse cultivation

To sample pure plant material, catch crops and wheat, which was the preceding crop on the studied field sites, were germinated and pre-cultured for 7 days on peat-based Substrate1 (Klasmann-Deilmann, Geeste, Germany). Plants received additional light (630–900 µmol s^−1^ m^−2^, 16 h d^−1^) and were grown at 20–22 °C. Afterwards plants were transferred to 1:1 (w/w) compost soil and peat-based Substrate2 (Klasmann-Deilmann, Geeste, Germany) and cultivated for 7 weeks at additional light (720–1080 µmol s^−1^ m^−2^, 16 h d^−1^) and 18–20 °C.

#### Field cultivation

Field experiments were conducted at two locations. At Asendorf in northern Germany (49 m above sea level (a.s.l), 52°45′48.4″N 9°01′24.3″E, mean annual temperature (MAT): 9.3 °C, mean annual precipitation (MAP): 751 mm) soils developed from shallow loess over glaciofluvial sand (>50 cm) and were classified as Stagnic-Cambisol (IUSS Working Group^44^). Soil texture was uniformly silt-loam. The southern German site Triesdorf (450 m a.s.l, 49°12′36.5″N 10°38′33.9″E, MAT: 8.7 °C, MAP: 674 mm) was characterized by larger substrate heterogeneity. Soil texture ranged from sandy-loam to sandy clay-loam. The Stagnic-Cambisols developed from shallow loess over *in-situ* weathered sandstone-claystone (Keuper).

Field trials were installed in autumns 2015 and 2016. Table 1 shows weather data during the vegetation periods. In a completely randomized block design with a row spacing of 12.5 cm, six variants were sown: Pure stands of each, mustard, phacelia, oat and clover, a mixture of these four species and fallow plots representing control variants. Considering differences in juvenile development among species and to obtain comparable shoot biomass in all treatments, sowing densities were adjusted as listed in Table 2. For the mixture, seeds of all species were mixed as indicated in Table 2 before sowing them together in one seed mixture. On fallow plots, weed growth was suppressed by glyphosate in Triesdorf, or by manual weeding in Asendorf.

### Sampling and processing of plant material and soil cores

At the end of the vegetation period (Triesdorf: 29.10.2015/2.11.2016, Asendorf: 27.10.2015/24.10.2016) areal shoot biomass per species was determined in three 100 × 100 cm microplots after drying (3 d, 80 °C). Depending on site and year, the species had reached the following developmental stages: Mustard - BBCH50-60, phacelia - BBCH30-60, oat - BBCH35-40, clover - BBCH25-30 (after^45^).

In each plot, three soil cores of 6 cm diameter and a depth of >70 cm were taken randomly in and between the sowing rows using an automated soil corer (Nordmeyer GEOTOOL, Berlin, Germany). Cores were cut into slices. Slices from 0–2, 8–12, 18–22, 28–32, 38–42, 48–52, 58–62 and 68–72 cm depth were deep-frozen for root biomass analysis, while slices from 10–20, 20–30 and 50–60 cm were taken for the determination of soil water and element contents.

### Water and elemental analysis of soil material

In soil samples, water content was determined gravimetrically after oven-drying (105 °C, 24 h). Soil pH and conductivity was measured potentiometrically (pH: CG 842, Schott Instruments, Mainz, Germany; conductivity: Condi 340i and Tetracon® electrode, Xylem Analytics, Weilheim, Germany) at a soil:water ratio of 1:2.5 (w/v). The mineral N (N_min_) content was determined as sum of NH_4_^+^ and NO_3_^−^. ~10 g of fresh soil material was extracted with 0.0125 M CaCl_2_ at 1:4 (w/v) soil:solution, filtered (qualitative, grade 3HW, Sartorius, Göttingen, Germany) and analysed for NH_4_^+^ and NO_3_^−^ using an autoanalyser (SAN-plus, Skalar Analytical, Breda, Netherlands). Results were corrected for initial water content and referred to g soil dry weight (DW).

Plant-available P was analysed by Mehlich3 (M3) extraction according to Ziadi and Tran^46^. Briefly, 3 g of dry soil were extracted with M3 at 1:10 (w/v) soil:solution by shaking for 5 min and filtered through M3-rinsed Whatman#42 filter paper. All extracts were analysed for M3-extraxtable P (P_ex_) by ICP-OES (Varian 725-ES, Palo Alto, USA).

### Root biomass quantification in soil slices

To describe rooting behaviour of different catch crops in soil samples from field plots, a DNA-based method was set up for (i) assigning root material to individual species and (ii) quantifying their root biomass.

(i) Primers were designed for species-specific amplification of the internal transcribed spacer (ITS) 1 or ITS2 regions^28^. To obtain ITS sequences, genomic DNA (gDNA) was extracted (see 4.5) from frozen-ground leaf material of greenhouse-grown catch crop species, wheat and of the indoor species *Spathiphyllum* spec. (see below). ITS1-5.8SrDNA-ITS2 cassettes were amplified with the universal primers ITS-A and ITS-B^47^ in 35 PCR cycles (denaturation: 30 s, 95 °C; annealing: 30 s, 55 °C; extension: 1 min, 72 °C; final extension: 10 min, 72 °C) using GoTaq® DNA Polymerase Kit (Promega, Mannheim, Germany). After gel electrophoresis, amplified fragments were gel-extracted using QIAquick Gel Extraction Kit (Quiagen, Hilden, Germany) and sequenced (MWG Eurofins, Ebersberg, Germany). Using geneious R6 software, version 8.0.4 (Biomatters, Auckland, New Zealand), primers were designed for amplification in one of the ITS regions. Species-specificity of the primers (Metabion International, Planegg/Steinkirchen, Germany) was tested by PCR as described above, using primer-specific annealing temperatures and subsequent gel electrophoresis (2% (w/v) agarose, 0.006% (v/v) ethidium bromide) of amplicons from catch crops and *Spathiphyllum*. For primer design and species-specific DNA amplification we assured that wheat ITS DNA was not amplified by primers designed for catch crop and *Spathiphyllum* ITS. Finally, primer sets listed in Table 5 were chosen for species discrimination in soil samples.

**Table 5:** Primers and annealing temperatures for species-specific discrimination of DNA in soil samples.

Species	Forward primer (5′–>3′)	Reverse primer (5′–>3′)	Annealing temperature	Amplification in region
Mustard	TTTCTTTGCTGATTCTGTGCCTG	CGAAGTACTGGCTGGGAACTTAA	65 °C	ITS1
Phacelia	GGTTGTTATCTCAACTCGCGTG	GGTCTATTCAGTCCCGGCAG	64 °C	ITS2
Oat	TAAACACGCTCCCAACCCCTTA	TCGGAGACACTGCGGTAAGTATAG	66 °C	ITS2
Clover	TGAATTAGTTTCAACACATAGGGTTGGTTC	GAGCAAATTTTAAATTCCTTGACGCATTCAG	63 °C	ITS1
*Spathiphyllum*	CTCTGTCTGCCTGCCTATTTGTT	GTCATTCAGACTTAAACTTGCGACG	62 °C	ITS1

(ii) Species-specific primers were used to set up qPCR-based standard curves with Ct-values obtained from DNA of a known amount of root dry material from individual species. For this purpose, individual plants from each catch crop species were grown in their pure stand, but above 20 µm mesh-sized nets, which were installed at 30 cm depth. At the end of the cultivation period, roots of plants cultivated above the mesh were carefully washed out. This allowed retrieving root material under agricultural conditions for the calibration of standard curves. Although samples from different catch crop species contained root material of different age, none of the four catch crop species had reached maturity (BBCH stages see above), allowing to assume that the degradation of root DNA was not considerably different among species.

**Figure 3:**
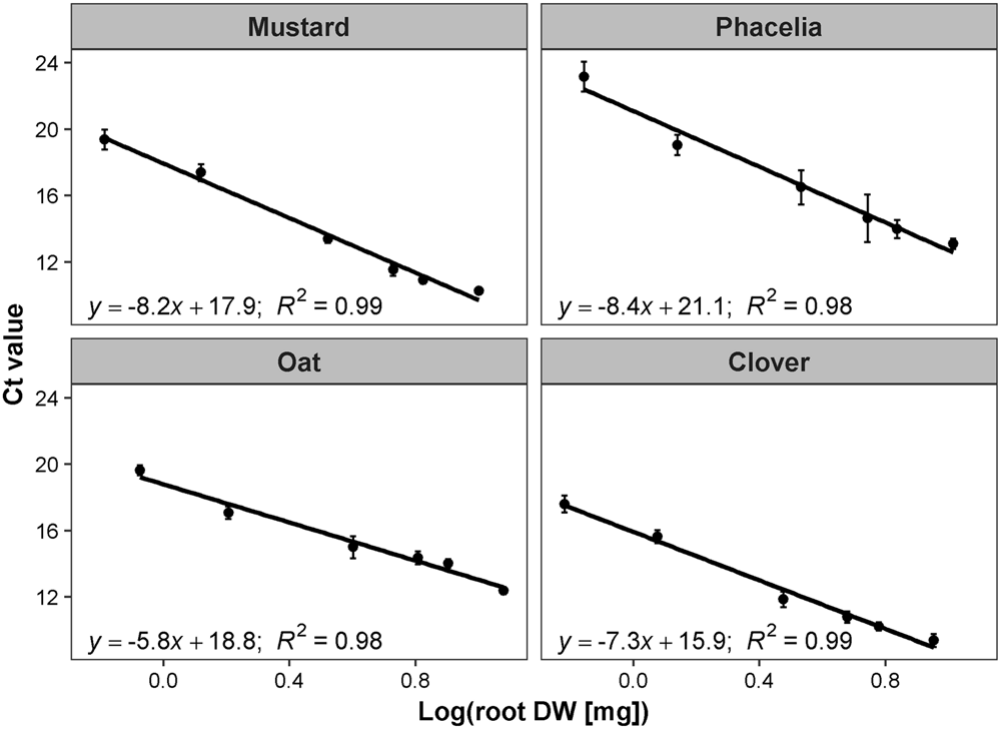
Ct-value standard curves for the determination of root DW of mustard, phacelia, oat and clover using qPCR. Circles show means ± s.d.; n = 9.

Roots from ten plants per species were ground, pooled and used for gDNA extraction (see 4.5) from 6× of either 10, 20, 50, 80, 100 or 150 mg root fresh weight (FW), while respective second aliquots were dried (80 °C, 2d) to determine DW. Additionally, gDNA was extracted from 20 × 20 mg frozen-ground leaf material of *Spathiphyllum* for later use as internal standard for DNA extraction efficiency (see below). Obtained gDNA samples were run in 44 qPCR cycles (denaturation: 10 s, 95 °C; annealing: 20 s, primer-specific temperature (Table 5), extension: 30 s, 72 °C) followed by a melt curve (CFX384 Touch Real-Time PCR Detection System, software: BioRad CFX Manager version 3.1; BioRad, Munich, Germany) with species-specific primers using iQ™ SYBR® Green Supermix (BioRad, Munich, Germany). Ct-values from every reaction were plotted against the logarithmic root DW corresponding to the deployed root FW for gDNA extraction. This provided Ct-value-based standard curves to determine root DW of each species in soil samples (Fig. 3).

Field-obtained soil slices were carefully washed with cold tap water through a 0.4 mm mesh-sized sieve to remove small soil particles rich in clay and organic matter and to reduce soil:root ratios. In pre-tests, washing lowered interference of gDNA extraction with soil organic substances and increased gDNA yield. Remaining sample material (roots and sand) was frozen-ground and ~600 mg were used for gDNA extraction following the protocol used for pure plant material to establish species-specific qPCR standard curves (see 4.5). Since an interference of DNA extraction with the remaining soil matrix could not be completely excluded, 20 mg of *Spathiphyllum* leaf material, natively occurring in South America, were added as internal standard to every 600 mg-soil sample prior to gDNA extraction to correct for extraction efficiency^28^. Every sample was run at the described qPCR conditions with species-specific primers for catch crops and *Spathiphyllum*. Using *Spathiphyllum* Ct-value differences between qPCRs on pure *Spathiphyllum* gDNA and on *Spathiphyllum* plus soil samples, DNA extraction efficiency was corrected. Using the Ct-value standard curves (Fig. 3), root DW of catch crops in different soil depths were calculated. Soil samples from fallow, processed in the same way, were used as control for present DNA stocks of each species in soil profiles of the field sites^28^. Taking weight per volume and water content in different depths into account, root biomass of individual species was related to soil volume within the vertical profile.

### Extraction of genomic DNA

gDNA was extracted following Murray and Thompson^48^. Buffer1 (2% (w/v) cetyl trimethylammonium bromide (CTAB), 100 mM Tris-HCl pH 8.0, 20 mM EDTA, 1.4 M NaCl, 0.5% (w/v) PVP), pre-heated to 60 °C, was added to ground plant material or root/sand mixtures (remaining from soil samples after washing), gently mixed and incubated (5 min, 70 °C). After adding dichloromethane, samples were centrifuged (208,000 *g*, room temperature). The recovered aqueous phase was mixed with the double volume of Buffer2 (2% (w/v) CTAB, 50 mM Tris-HCl pH 8.0, 10 mM EDTA, 0.25% (w/v) PVP). CTAB-bound DNA was precipitated overnight and spun down (208,000 *g*). DNA-CTAB complexes in the pellet were dissolved using 1 M NaCl, and DNA was precipitated with isopropanol overnight. Again, DNA was spun down and washed in 80% ethanol. Finally, dried DNA pellets were dissolved in TE-buffer (10 mM Tris pH 8.0, 0.1 mM EDTA pH 8.0, 0.00075% (w/v) RNAse A; Thermo Fisher Scientific, Schwerte, Germany).

### Statistical analysis

For statistical analyses the program SigmaPlot 11.0 (Systat Software, Erkrath, Germany) was used. All tests were performed at a 95% confidence level.

Gaussian distribution was checked using the Shapiro-Wilk test. If data followed normal distribution multiple comparison analysis was done by ANOVA using Tukey’s test as post-hoc test. Correlations of normal distributed data were calculated by Pearson product moment correlation. Nonparametric tests were chosen for data not following Gaussian distribution: Kruskal-Wallis one-way ANOVA on ranks with Tukey’s test for multiple comparisons and Spearman rank order for correlations.

## Supplementary information

Supplementary Information

## Data Availability

The datasets generated and/or analysed during the current study are available in the e!DAL repository under 10.5447/IPK/2019/12.
