# Author Correction: Interspecific competition among catch crops modifies vertical root biomass distribution and nitrate scavenging in soils

**DOI:** 10.1038/s41598-020-61543-9

**Published:** 2020-03-10

**Authors:** Diana Heuermann, Norman Gentsch, Jens Boy, Dörte Schweneker, Ulf Feuerstein, Jonas Groß, Bernhard Bauer, Georg Guggenberger, Nicolaus von Wirén

**Affiliations:** 1 Molecular Plant Nutrition, Leibniz Institute of Plant Genetics and Crop Plant Research Gatersleben, Corrensstraße 3, 06466 Stadt Seeland, Germany; 2 Institute of Soil Science, Leibniz Universität Hannover, Herrenhäuser Straße 2, 30419 Hannover, Germany; 3 Deutsche Saatveredelung AG, Steimker Weg 7, 27330 Asendorf, Germany; 4 Crop Production and Crop Protection, Hochschule Weihenstephan-Triesdorf, Steingruberstraße 2, 91746 Weidenbach, Germany; 5 Present Address: Kuratorium für Technik und Bauwesen in der Landwirtschaft e.V., Bartningstraße 49, 64289 Darmstadt, Germany

Correction to: *Scientific Reports* 10.1038/s41598-019-48060-0, published online 08 August 2019

In Figure 1, the plots of ‘Asendorf 2015’, ‘Asendorf 2016’ and ‘Triesdorf 2016’ have incorrect Y-axis scale measurements. The correct Figure 1 appears below.

**Figure 1:**
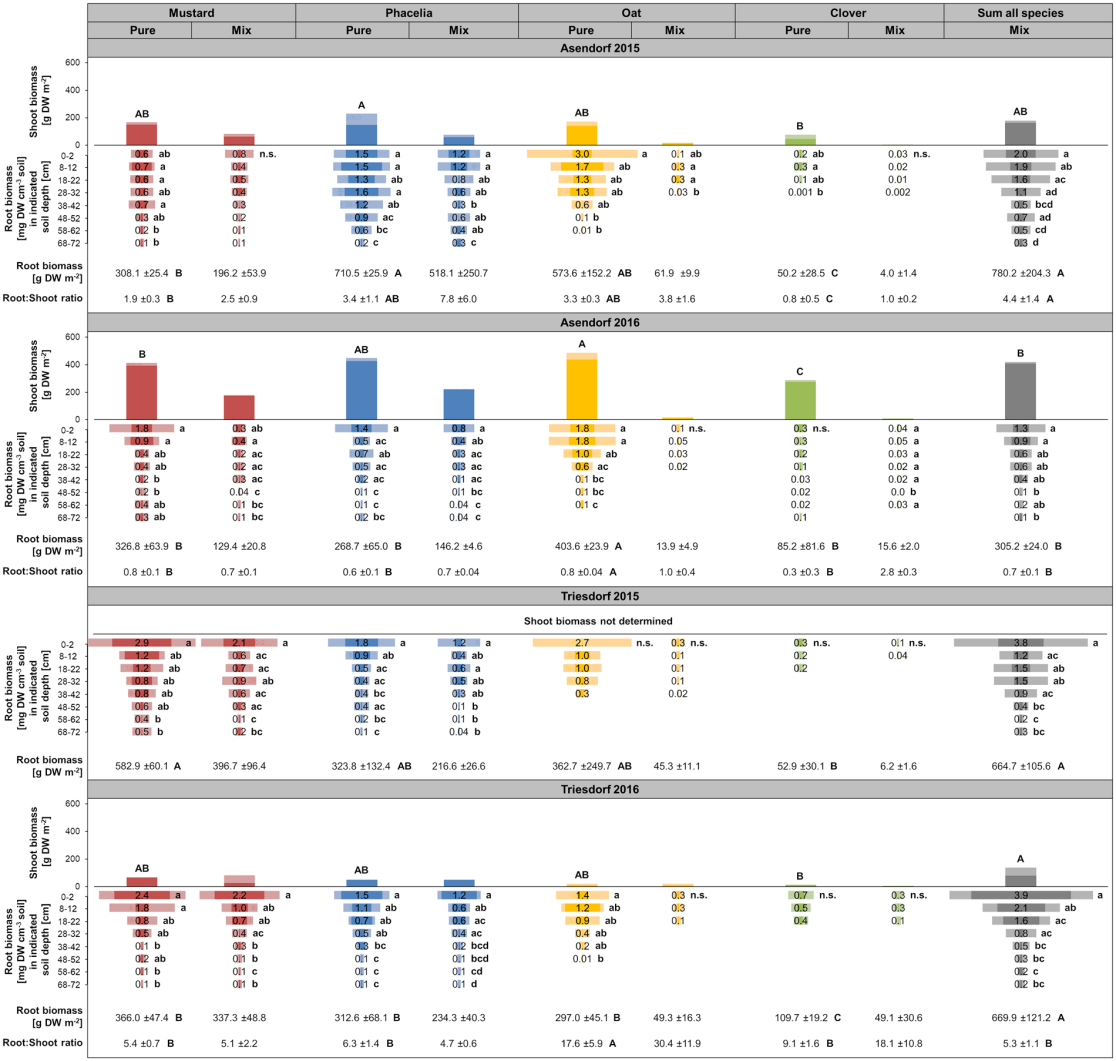
.

