# Supplementary material for: Interspecific competition among catch crops modifies vertical root biomass distribution and nitrate scavenging in soils
Source: Sci Rep. 2019 Aug 8;9:11531. doi: 10.1038/s41598-019-48060-0 (PMC6687801; doi:10.1038/s41598-019-48060-0)
Supplement: Supplementary file 1 — Supplementary Information [file 41598_2019_48060_MOESM1_ESM.pdf]

Supplementary information

Interspecific competition among catch crops modifies vertical root biomass distribution and nitrate scavenging in soils

Diana Heuermann[1], Norman Gentsch[2], Jens Boy[2], Dörte Schweneker[3], Ulf Feuerstein[3], Jonas Groß[4,5], Bernhard Bauer[4], Georg Guggenberger[2] and Nicolaus von Wirén[1*]

[1] Molecular Plant Nutrition, Leibniz Institute of Plant Genetics and Crop Plant Research Gatersleben, Corrensstraße 3, 06466 Stadt Seeland, Germany

[2] Institute of Soil Science, Leibniz Universität Hannover, Herrenhäuser Straße 2, 30419 Hannover, Germany

[3] Deutsche Saatveredelung AG, Steimker Weg 7, 27330 Asendorf, Germany

[4] Crop Production and Crop Protection, Hochschule Weihenstephan-Triesdorf, Steingruberstraße 2, 91746 Weidenbach, Germany

[5] Present address: Kuratorium für Technik und Bauwesen in der Landwirtschaft e.V., Bartningstraße 49, 64289 Darmstadt, Germany

* Corresponding author: Nicolaus von Wirén (vonwiren@ipk-gatersleben.de;

+49 39482-5602)

Supplementary Table S-1: Soil water content at the experimental stations Asendorf and Triesdorf in 2015 and 2016. Table shows means ±SD; n=3. Vertical letters: Differences in soil water content within one depth among catch crop variants according to Tukey´s test on ranks at $p<0.05$; ns.=not significant.

**Catch crop variant**	**Soil water content [%]**			
	**Asendorf**		**Triesdorf**	
	**2015**	**2016**	**2015**	**2016**
0-10 cm				
Control	23.0 ±2.4 [ns.]	9.5 ±2.2 [ns.]	17.4 ±0.6 [ns.]	11.6 ±0.9 [ns.]
Mustard	22.0 ±1.4	11.2 ±1.0	16.5 ±0.9	11.8 ±0.2
Phacelia	21.5 ±0.8	9.6 ±3.3	15.4 ±0.6	12.2 ±0.5
Oat	21.8 ±0.6	10.6 ±1.7	15.4 ±0.2	11.9 ±0.7
Clover	22.3 ±1.0	8.8 ±1.8	16.8 ±0.8	12.2 ±1.3
Mix	22.7 ±2.0	11.6 ±0.6	15.3 ±0.3	11.6 ±0.8
20-30 cm				
Control	19.1 ±3.4 [ns.]	6.9 ±2.2 [ns.]	12.1 ±0.9 [ns.]	9.0 ±0.5 [ns.]
Mustard	18.6 ±2.4	6.1 ±0.2	9.1 ±2.6	14.4 ±4.8
Phacelia	19.2 ±0.6	6.7 ±0.5	8.5 ±0.9	9.1 ±0.4
Oat	18.4 ±0.3	6.5 ±0.8	11.0 ±4.5	8.5 ±0.2
Clover	19.1 ±0.5	6.2 ±0.7	10.5 ±1.5	9.2 ±0.3
Mix	20.1 ±1.9	6.5 ±0.4	7.9 ±1.3	9.1 ±0.7
50-60 cm				
Control	17.2 ±2.9 [ns.]	8.8 ±0.4 [ns.]	6.1 ±1.0 [ns.]	7.5 ±2.8 [ns.]
Mustard	15.8 ±1.1	6.8 ±0.6	8.8 ±4.3	9.1 ±2.8
Phacelia	15.3 ±2.1	8.0 ±1.3	6.8 ±1.3	8.4 ±2.6
Oat	15.3 ±2.5	7.8 ±1.2	8.3 ±1.7	9.1 ±4.8
Clover	16.1 ±2.8	6.6 ±1.5	6.6 ±0.8	6.4 ±2.0
Mix	15.9 ±2.6	7.8 ±1.3	9.7 ±4.7	6.5 ±2.6
